# Matrons in Council: VI. Modern Ideas Needed, Though Not Favoured in Our Hospitals

**Published:** 1917-09-15

**Authors:** 


### THE MATRONS' AND SISTERS' DEPARTMENT.

### MATRONS IN COUNCIL.

### VI. Modern Ideas Needed, Though not Favoured in our Hospitals.

The points raised in "Sparks from a Nurse's Anvil" will be appreciated by many members of the nursing profession. Many reforms are needed, and will have to be brought about very soon if a different class of candidate is to be attracted to the profession. Training-schools will have to be reconstructed in accordance with the reconstruction of the rest of the world.

To the lay person who is allowed an insight into hospital life, the life appears narrow, old-fashioned, and bound down by rules and conventionalities which in these days of emancipation are not tolerated. Speaking generally, modern ideas have not been introduced into our hospitals, neither are they favoured, and this is quite probably the reason why educated women are not attracted to the profession when so many other professions are open to them which give greater opportunities for retaining individuality, and offer more adequate remuneration on the completion of training.

Florence Nightingale said: "Nursing is an art, and if it is to be made an art, requires as exclusive a devotion, as hard a preparation as any painter's or sculptor's work, for what is the having to do with dead canvas or cold marble compared with having to do with the living body, the temple of God's Spirit? It is one of the fine arts—I had almost said the finest of the fine arts. Probably no person ever did that well which he did only for money. Certainly no person ever did that well which he did not work at as hard as if he did it solely for money. If by amateurs in art or in nursing are meant those who take it up for play, it is not art at all, it is not nursing at all. You never yet made an artist by paying him well. But—an artist ought to be well paid."

Although there has been considerable divergence from Florence Nightingale's idea, the ideal still remains.

Nursing for pure love of the work, in these days of competition, hurry, and bustle, attracts a very small minority of the candidates who offer themselves for training. This is not altogether due to lack of love and sympathy for suffering humanity, but to the matter-of-fact, unsentimental and perhaps hypocritical age in which we live; any underlying love and sympathy which may decide the choice of a profession is relegated to the background as "mere sentiment."

If Florence Nightingale's idea that nursing should be in the hands of educated women is again to be realised, new traditions will have to be created, and the door of the profession closed to the uneducated, for in no sphere of life, even in these democratic days, do the educated and uneducated work amicably together, except, of course, in a time of national necessity like the present. In order to ensure a uniform educational standard it would seem advisable for all candidates to pass a preliminary examination in general knowledge before being accepted by the hospital authorities on probation.

The combination of the quality of ideal and the quality of performance should be possible if the highest ideals are constantly upheld and practised by the matron and her sisters, and the highest standard of efficiency maintained at all times.

More thought and time should be devoted to the teaching of practical work. In the majority of training-schools the probationer is taught a scrap of practical work during the rush and bustle of the day's routine, when details are hurriedly explained to her; while in some she is left to find things out for herself. Owing to the small number of staff in each ward (even in pre-war days) this is unavoidable, and the remedy seems to lie in the provision of more nurses, thereby lessening the number of beds allotted to each one, and leaving the sister free for supervision and teaching. Only those women who live up to the highest ideals, are thoroughly and efficiently practical, and have the gift of imparting knowledge, should be granted the higher appointments. Trained workers must adopt the attitude of teacher and pupil, rather than that of mistress and servant, which is the attitude of many of the present-day workers. They must enter into the hearts and sympathies of their pupils, enter into their recreations, and talk to them as women to women, and give opportunities for social intercourse outside the hospital. This could be done by the right women in the higher positions without any loss of dignity or slackness of discipline, which, of course, must be maintained if the hospital is to work smoothly. Many of the rules and conventionalities which are decidedly narrow could be relaxed or dispensed with, without fear of undue advantage being taken, always provided that the educated woman with the trained and disciplined mind, a state brought about by education, entered the ranks of the profession. Uneducated people take advantage more readily, owing to the lack of mental disciplinary training, which necessitates the rigid adherence to rules in order to maintain discipline.

A reform sorely needed is a preliminary training school where probationers would be taught the rudiments of nursing, bedmaking, preparation and cooking of food, hygiene, and cleaning, which latter is absolutely essential to a nurse's training, but when taught in the wards is apt to be placed before the care of the patients. If such training covered a period of from two to three months, the probationer could on entrance into the hospital devote her

☛ Previous Articles appeared in The Hospital of August 4, 11, 18, 25, and September 1.

time to learning how to nurse instead of how to clean and scrub. This scheme would, of course, necessitate a larger staff of wardmaids, but the advantages derived therefrom would tend to make for a higher standard of nursing. In a few of the larger schools the preliminary training school has been established; one can quite well see that such a scheme has disadvantages when suggested in connection with a small school where entries are not numerous; but it might be possible for several small training-schools to form a centre, although this, again, would have its disadvantages, unless the same method of training prevailed in all the hospitals.

With regard to the Military Nursing Service, much of the conservatism which now exists and permeates through the various members of the service, thereby causing a feeling of separateness between the Army and civilian nurses, might be abolished if the Matron-in-Chief, with some of her staff, would meet democratic members of the nursing world and discuss with them in a broad-minded way their views on the present-day nursing question. The conventions and red-tape by which the members of the service are bound down do not tend to make for harmonious working together.

One does not altogether approve of all these divergences from the ideas of Florence Nightingale, who had little sympathy with the emancipated woman; but the question of reconstruction has to be faced, and in the near future, if the right kind of woman is to be attracted to the profession.

Some of the reforms necessary are the adoption of broader-minded views by those holding the higher appointments, relaxation of rules, less restraint, less conventionality, more adequate remuneration on completion of training, a shorter working day, more opportunities for recreation and social intercourse, time set apart for lectures and study (which at the present time are usually counted as "off duty"), and a uniform educational standard; all trained workers regarded as equals, as far as is compatible with the maintenance of discipline; and at all times should the highest ideals and a high standard of efficiency be upheld and maintained.

If these reforms could be carried into effect the profession would undoubtedly be more attractive to the woman having a trained and disciplined mind, who ought at all times to seek to uphold and raise the reputation of her *Alma Mater*, and not take undue advantage of the many facilities offered. She would ask herself occasionally if she were living up to her profession, and would remember that a nurse's life is above all a moral and practical life—a life not of show, but of practical action, and would strive by the best that was in her to act up to her profession, and to rise continually to a higher level of thought and practice, character and dutifulness.

